# Repository Corticotropin Therapy for Refractory Noninfectious Inflammatory Ocular Diseases

**DOI:** 10.3390/jcm14217785

**Published:** 2025-11-02

**Authors:** Christian Nieves-Ríos, Ricardo A. Murati Calderon, Armando L. Oliver

**Affiliations:** Department of Ophthalmology, School of Medicine, University of Puerto Rico Medical Sciences Campus, San Juan, PR 00936, USA; christian.nieves10@upr.edu (C.N.-R.); armando.oliver@upr.edu (A.L.O.)

**Keywords:** adrenocorticotropic hormone, noninfectious, uveitis, scleritis, cicatricial pemphigoid

## Abstract

**Objectives**: To describe the outcomes of patients with refractory noninfectious ocular inflammatory diseases who underwent treatment with repository corticotropin injection (RCI). **Methods**: A retrospective cohort study was conducted. Patients who failed treatment with corticosteroids and were subsequently treated with RCI were included. Primary outcome measures were intraocular inflammatory activity, intraocular pressure (IOP), and the development of complications. **Results**: A total of 19 eyes from 10 patients were included. Most of the patients were women (70.0%) and the median age at presentation was 49.0 years (30.0–84.0 years). The ocular diagnoses were anterior/intermediate uveitis, intermediate/posterior uveitis, panuveitis, ocular cicatricial pemphigoid, and anterior scleritis. Seventeen (89.5%) eyes had active disease. The median duration of RCI treatment was 16.0 months (6.0–28.0 months). Nine (90.0%) patients, representing 17 (89.5%) eyes, achieved disease inactivity and remained quiescent at the last visit. No patient was on systemic corticosteroids at the last evaluation. The mean IOP was lower under RCI than corticosteroid at one month (16.0 mmHg ± 6.1 vs. 20.8 mmHg ± 9.5, *p* = 0.033) and four months (15.6 mmHg ± 2.9 vs. 17.8 mmHg ± 3.7, *p* = 0.046); however, the overall difference was not significant (16.2 mmHg ± 1.1 vs. 17.3 mmHg ± 1.8, *p* = 0.057). Incidence rates were the highest for posterior subcapsular cataracts (44.4% per eye-year). Relative risk analysis (RR) showed a 40.0% risk reduction for cystoid macular edema (RR = 0.60, *p* = 0.054). **Conclusions**: RCI may be an alternative treatment for refractory noninfectious ocular inflammatory diseases in patients who have failed treatment with corticosteroids.

## 1. Introduction

Noninfectious ocular inflammatory diseases, including uveitis, ocular cicatricial pemphigoid (OCP), and scleritis, have been associated with significant visual morbidity and blindness [1]. The main objective of treatment regimens is to control intraocular inflammation and preserve vision [1,2]. For many years, corticosteroids have been considered the mainstay treatment for inflammatory eye diseases [1,3]. However, not infrequently, there are instances in which corticosteroid monotherapy is no longer indicated and treatment with immunosuppressive agents is warranted [1]. Unfortunately, many of these agents are potentially toxic and have been associated with serious systemic adverse effects, thereby limiting their implementation [4]. Therefore, alternative agents for treating inflammatory ocular pathologies—including repository corticotropin injection (RCI)—have been studied.

RCI is a naturally sourced complex mixture of adrenocorticotropic hormone (ACTH) analogs and other pituitary peptides [5,6,7]. ACTH is a member of the melanocortins, a group of peptides derived from proopiomelanocortin [8,9]. This molecule exerts anti-inflammatory and immunomodulatory effects via two independent mechanisms [10]. The first mechanism works via the ACTH-induced activation of melanocortin receptor 2, which stimulates endogenous cortisol production by the adrenal cortex, resulting in a generalized reduction in the inflammatory response [10]. The second mechanism acts on melanocortin receptors 1, 3, and 5, which are expressed by immune cells, such as macrophages, neutrophils, lymphocytes, and endothelial cells, reducing leukocyte infiltration, inhibiting cytokine production, and increasing phagocytosis [10]. In ocular tissues, these receptors are expressed on the retinal pigment epithelium, choroidal endothelium, and infiltrating leukocytes, and their activation helps preserve the blood–retina barrier while limiting leukocyte trafficking and pro-inflammatory cytokine release [6,11]. This is a localized and finely regulated anti-inflammatory circuit independent of the hypothalamic–pituitary–adrenal axis [10]. These mechanisms of immune regulation serve as the basis for disease control and anti-inflammatory effects in non-infectious ocular inflammatory diseases.

RCI has received regulatory approval for the treatment of ophthalmic diseases but is not widely included in or addressed by guidelines for the management of ocular inflammatory diseases [5]. Nonetheless, the steroid-independent, anti-inflammatory mechanism of RCI may be a suitable alternative for disease control that, notably, often spares patients from steroidogenic side effects [6,10,12]. This distinct mechanism has led to increasing interest in evaluating the effects of corticotropin hormone on the eyes [8]. However, the literature on RCI for ocular inflammatory diseases is scarce [4,13,14,15,16]. The purpose of this study was to explore the therapeutic effects of RCI in patients with refractory noninfectious inflammatory eye diseases.

## 2. Materials and Methods

A retrospective cohort study was conducted. All the patients with a diagnosis of noninfectious ocular inflammatory disease were identified. Patients who failed the initial treatment with corticosteroids, with or without an immunomodulatory agent (IMA), and subsequently underwent treatment with RCI (Acthar Gel, Mallinckrodt, Dublin, Ireland, 80 U/mL, subcutaneously, twice weekly) were included. Treatment failure was defined as either an inadequate response to corticosteroids or the onset of unacceptable or intolerable side effects. A minimum follow-up period of 1 year was used. This study was approved by the Institutional Review Board of the University of Puerto Rico, complied with the Health Insurance Portability and Accountability Act of 1996, and adhered to the tenets of the Declaration of Helsinki.

The electronic medical records from three uveitis clinics in Puerto Rico (Medical Services Administration Ophthalmology Clinic, San Francisco Ophthalmology Group, and Instituto de Ojos y Piel) were reviewed, and data from January 2018 through January 2022 were collected. The clinical information gathered included sociodemographic factors (age and sex), comorbidities, medical and ocular histories, medication regimen, ophthalmological examinations (visual acuity, intraocular pressure [IOP], slit-lamp examination), the grade of inflammation in the anterior and posterior segments [17], and the presence of ocular or systemic complications. Eye-specific clinical data were collected for both eyes at every clinic visit.

The mean IOPs were compared for the same patients during two different treatment protocols, the six months on corticosteroids prior to RCI vs. the initial six months after starting RCI therapy. In addition, the incidence rates (IRs) of the ophthalmological complications, including ocular hypertension, cystoid macular edema (CME), epiretinal membrane (ERM), and posterior subcapsular cataracts, was evaluated for complication-free eyes at baseline. Finally, the effect of RCI on the development of ocular complications was also assessed.

Descriptive analyses such as means, medians, percentages, interquartile range (IQR), and ranges were conducted using Microsoft Excel. R software (Version 4.1.1) was used for statistical tests. A bootstrap Welch two-sample *t* test was used to compare IOP between the corticosteroid and the RCI treatment groups, using the Mkinfer package with 10,000 repetitions. Both an overall statistic as well as monthly tests were conducted.

The IRs for vision loss and ocular complications were estimated per eye-year by dividing the number of events by the total of the group’s individual time at risk of the event. Confidence intervals (CIs) were obtained using the epiR package in R. For the relative risk (RR), visits in which patients were not receiving RCI served as the control group for the analysis. The epitools package was used to calculate the estimated RR, the CIs, and *p*-value.

## 3. Results

### 3.1. Study Population

A total of 10 patients and 19 eyes were included in the study (Table 1). The median follow-up was 40.0 months (IQR = 15.0). Most of the patients were women (70.0%), and the median age at presentation was 49.0 years (IQR = 25.5). The ocular diagnoses included anterior/intermediate uveitis in four patients (40.0%) and eight eyes (42.1%), intermediate/posterior uveitis in two patients (20.0%) and four eyes (21.1%), panuveitis in one patient (10.0%) and two eyes (10.5%), OCP in two patients (20.0%) and four eyes (21.1%), and anterior scleritis in one patient (10.0%) and one eye (5.3%).

### 3.2. Baseline Clinical Presentation

The baseline clinical characteristics at the time of starting RCI are summarized in Table 2. Seventeen (89.5%) of the affected eyes had active disease. The patient with bilateral inactivity was started on RCI due to refractory, visually disabling CME. All 10 patients had previously failed to respond to at least one IMA. Table 3 shows the previously failed IMA for each patient before starting RCI.

### 3.3. Outcomes and Follow-Ups

The median duration of RCI treatment was 16.0 months (IQR = 12.0). Nine (90.0%) patients, representing 17 (89.5%) eyes, achieved sustained disease inactivity for at least three months. All patients were on at least one IMA at the time of starting RCI. There were no modifications to the IMA regimen at the start of RCI or during the follow-up period, except for two patients who were able to discontinue IMAs due to disease inactivity and maintain disease quiescence on RCI monotherapy. At the last visit, no patient was on systemic corticosteroids, though 80.0% remained on the initial RCI dosage (twice weekly) for adequate disease control. Treatment was discontinued in one patient due to recurrent ocular hypertension refractory to treatment. No other local or systemic adverse effects were identified.

### 3.4. Mean Intraocular Pressure During Treatment

**Figure 1:**
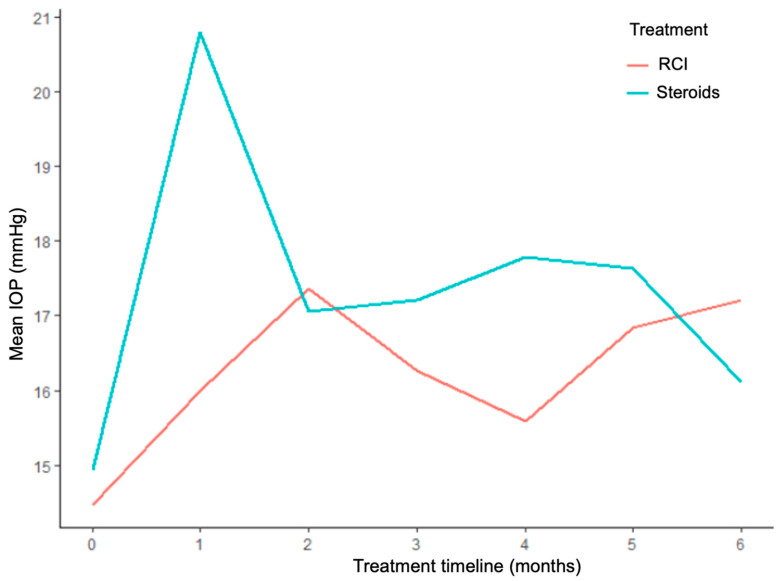
Comparison of mean intraocular pressure between repository corticotropin injection and corticosteroid.

In terms of IOP, Figure 1 compares the monthly fluctuations in mean IOP during a six-month period for the same patients under corticosteroids and repository corticotropin therapy. The greatest difference between treatments occurred at the one-month and four-month periods, when mean pressures under RCI (16.0 mmHg ± 6.1 and 15.6 mmHg ± 2.9, respectively) were significantly lower compared with corticosteroids (20.8 mmHg ± 9.5 and 17.8 mmHg ± 3.7, respectively) (*p* = 0.033 and 0.046, respectively). The overall difference throughout the six-month period between repository corticotropin (16.2 mmHg ± 1.1) and corticosteroid therapy (17.3 mmHg ± 1.8) did not achieve statistical significance (*p* = 0.057). Additionally, six (60.0%) patients (12 of the 19 eyes) required IOP-lowering medications during the study period. The mean number of medications for IOP control was 1.8 ± 0.8 on corticosteroids and 1.3 ± 1.0 on ACTH gel treatment.

### 3.5. Incidence of Vision Loss and Ocular Complications

The loss of vision was analyzed across different thresholds of visual acuity. The rates of visual acuity of 20/70 or worse and 20/200 or worse were 11.4% per eye-year and 8.0% per eye-year, respectively. The IRs of ocular hypertension (IOP > 21 mmHg) and CME were 9.8% per eye-year and 6.5% per eye-year, respectively. Finally, the rates of newly diagnosed ERM and cataracts were 9.5% per eye-year and 44.4% per eye-year, respectively (Table 4).

### 3.6. Association with Vision Loss and Complications

All the patients had follow-up appointments, received corticosteroids, and subsequently switched to a standard dose of RCI. Immunosuppressive therapy was required for all the patients at some point during the follow-up period. The follow-up encounters in which patients received corticosteroids but not repository corticotropin therapy functioned as the control group for the analysis. Table 5 summarizes the RRs of visual impairment and ocular complications. The results showed that RCI was associated with a 40.0% risk reduction in the development of CME; however, this result did not achieve statistical significance (RR = 0.60, *p* = 0.054).

## 4. Discussion

Our study suggests that RCI may be considered an alternative or adjunctive treatment for refractory noninfectious ocular inflammatory diseases in patients who have failed treatment with corticosteroids. Most of the patients achieved disease quiescence and remained inactive as of their last visit. Moreover, systemic corticosteroids were successfully reduced and eventually discontinued in all the patients, which suggests a possible role for RCI as a steroid-sparing agent. Disease activity persisted in only one case, which was characterized by HLA-A29-associated bilateral birdshot retinochoroidopathy. Marked improvement in vitreous inflammation was noted, though there was evidence of active chorioretinitis on imaging. However, this result was not unexpected, given that long-term therapy is usually required for this condition [5].

Ocular hypertension has been well described in the setting of corticosteroid use. Given the steroidogenic effect of RCI, an elevation in IOP may be expected [7]. In our study, 90.0% of the patients maintained a stable IOP after starting RCI. A possible explanation for this finding could be that the steroid responders were already on IOP-lowering medications before switching from corticosteroids to RCI. Nonetheless, at last visit, 50.0% of the steroid responders reduced or discontinued IOP-lowering medications while still maintaining disease quiescence under RCI. These findings are in agreement with those of a previous report in which no significant or concerning increase in IOP was observed in patients treated with RCI for noninfectious retinal vasculitis [14], suggesting that RCI may be a potential alternative treatment for steroid responders.

Sustained intraocular inflammation can result in significant visual morbidity and the development of ophthalmological complications, including cataracts [1,18]. Most of the patients in our study maintained stable visual acuity during the follow-up period. However, our results show slightly higher incidences of ocular complications than have been revealed by other reports [19]. This might be explained by the small sample size of patients in our study, particularly in the incidence of cataracts, of which only half of the eyes were at risk during the study period. Nonetheless, the chronic, refractory, and progressive nature of the cases included in our cohort may have contributed to the development of the observed complications.

Macular edema is one of the most common causes of vision loss in patients with intraocular inflammatory disease [20,21]. Generally, a stepwise therapeutic approach is used, though managing chronic and persistent cases can be challenging [22]. In 2016, Agarwal et al. described a case of recurrent noninfectious panuveitis and macular edema, with both marked improvement in inflammation and resolution of the edema after treatment with RCI [13]. In line with that, our results demonstrated a 40% risk reduction for CME in patients using RCI. Though this result did not achieve statistical significance (*p* = 0.054), it may offer clinically significant insight into the therapeutic role of RCI for patients with refractory CME.

Currently, RCI is advertised as treatment for severe acute and chronic allergic and inflammatory processes involving the eye and its adnexa [7]. Previous reports have evaluated the use of RCI for noninfectious uveitis, suggesting that this treatment may play a role in managing refractory and steroid-dependent cases and in treating patients who do not respond to or cannot tolerate other immunomodulatory therapies [13,23]. A recent Delphi study reached consensus that RCI may be considered for posterior and intermediate uveitis when other therapies are ineffective or not tolerated [5]. Furthermore, the panelists agreed to consider RCI as a treatment option for patients with OCP [5]. This consideration was supported by recent literature describing significant improvement in ocular surface inflammation, long-term control of disease activity, and no serious adverse events [4,15,24,25]. As of the writing of this manuscript, there is an ongoing multicenter, randomized clinical trial to evaluate the potential role of RCI in the management of noninfectious scleritis [26]. However, international studies evaluating the use of RCI in ocular diseases are scarce, which may reflect a limited availability of the medication.

There were various limitations to our study. The small sample size and limited study period may affect the statistical power and generalizability of the findings. This was a retrospective study with varied etiologies; therefore, it is not possible to establish a direct cause-and-effect correlation between RCI and the ophthalmological findings for each specific disease. A referral bias may exist due to the clinic’s affiliation with a tertiary care academic medical center, which may result in the overrepresentation of the most severe and refractory cases. Due to the inclusion criteria and disease severity, a cohort of patients without medications was not included; therefore, determining the effect of RCI alone may not be possible. Lastly, RCI is not readily available in all countries, which limits its applicability. Additional prospective and international studies with larger cohorts and longer follow-up intervals are warranted to confirm these results and evaluate the indications of RCI for noninfectious uveitides.

Our manuscript describes the presentation and outcomes of 10 patients who underwent treatment with RCI for refractory ocular inflammatory diseases. In addition, analyses describing the IOP changes under RCI therapy, the incidence of vision loss and of ocular complications, and the association of all three with the treatment were presented. Our results suggest that RCI may serve as an alternative or adjunctive therapy for recurrent noninfectious ocular inflammatory diseases that are refractory to standard therapy.

## Figures and Tables

**Table 1:** Sociodemographic factors.

Characteristic	A/I Uveitis	I/P Uveitis	Panuveitis	OCP	Scleritis	Total
Number of patients	4	2	1	2	1	10
Median age (years)	70 (48–84)	40 (30–49)	41 (no range)	58 (47–68)	44 (no range)	49 (30–84)
Sex (female)	3	2	1	0	1	7 (70%)
Diabetes mellitus	0	0	1	0	0	1 (10%)
Hypertension	3	0	0	2	0	5 (50%)
Dyslipidemia	2	0	0	2	0	4 (40%)
Atherosclerotic heart disease	1	0	0	1	0	2 (20%)
Hypothyroidism	1	1	0	0	0	2 (20%)

A/I: anterior/intermediate; I/P: intermediate/posterior; OCP: ocular cicatricial pemphigoid.

**Table 2:** Baseline characteristics at the time of starting repository corticotropin injection.

Characteristic	A/I Uveitis	I/P Uveitis	Panuveitis	OCP	Scleritis	Total
*Patient specific*						
Number of patients	4	2	1	2	1	10
Bilateral inflammation	4	2	1	2	0	9 (90%)
*Eye-specific*						
Number of affected eyes	8	4	2	4	1	19
Overall activity						
Active	6	4	2	4	1	17 (89%)
Conjunctival injection	0	1	0	4	0	5 (26%)
Scleral injection	0	0	0	0	1	1 (5%)
Anterior chamber cells						
No cells	2	4	0	4	1	11 (58%)
1.0+	2	0	2	0	0	4 (21%)
≥2+	4	0	0	0	0	4 (21%)
Vitreous cells						
No cells	4	2	0	4	1	11 (58%)
1.0+	2	2	0	0	0	4 (21%)
≥2+	2	0	2	0	0	4 (21%)
Active retinal vasculitis	0	4	0	0	0	4 (21%)
Active choroidal lesion	0	4	2	0	0	6 (32%)

**Table 3:** Previously failed immunomodulatory agents before starting repository corticotropin injection treatment.

Patient	Diagnosis	Tx#1	D (mg)	F	DTx (Months)	Tx#2	D (mg)	F	DTx (Months)	Tx#3	D (mg)	F	DTx (Months)
1	OCP	MycMof	1500	BID	6	Dap	100	QD	16	Chloram	12	QD	5
2	Panuveitis	MTX	25	Once/week	6	Cyclospo	200	BID	3	-	-	-	-
3	Scleritis	MycMof	1500	BID	4	MTX	25	Once/week	3	-	-	-	-
4	A/I	MTX	25	Once/week	3	Dupi	300	Once/week	23	-	-	-	-
5	A/I	MycMof	1500	BID	31	-	-	-	-	-	-	-	-
6	I/P	MycMof	1500	BID	12	Adali	40	Once/two weeks	8	-	-	-	-
7	I/P	MycMof	1500	BID	24	-	-	-	-	-	-	-	-
8	A/I	MycMof	1500	BID	17	MTX	25	Once/week	12	-	-	-	-
9	A/I	MycMof	1500	BID	22	-	-	-	-	-	-	-	-
10	OCP	MycMof	1500	BID	12	Dap	100	QD	27	Cycloph	500	Once/month	2

A/I: anterior/intermediate; Adali: adalimumab; BID: twice a day; Chloram: chroambucil; Cycloph: cyclophosphamide; Cyclospo: cyclosporine; D: dosage; Dap: dapsone; DTx: duration of treatment; Dupi: dupilumab; F: frequency; I/P: intermediate/posterior; MTX: methotrexate; MycMof: mycophenolate mofetil; OCP: ocular cicatricial pemphigoid; QD: once a day; Tx: treatment.

**Table 4:** Incidence rates of vision loss and ocular complications in ocular inflammatory diseases.

Event	Eyes (n/N) *	Rate per Eye-Year at Risk (95% CI)
VA 20/70 or worse	5/17	0.11 (0.04, 0.27)
VA 20/200 or worse	4/17	0.08 (0.02, 0.21)
Ocular hypertension	5/19	0.10 (0.03, 0.23)
Cystoid macular edema	2/11	0.07 (0.01, 0.23)
Epiretinal membrane	4/16	0.10 (0.03, 0.24)
Cataract	4/7	0.44 (0.12, 1.14)

* Number of events/number of eyes at risk; VA: visual acuity.

**Table 5:** Association of repository corticotropin injection and vision loss or ocular complications in ocular inflammatory diseases.

Event	Relative Risk (95% CI)	*p*-Value
VA 20/70 or worse	1.00 (0.65, 1.54)	1.000
VA 20/200 or worse	0.94 (0.73, 1.21)	0.670
Ocular hypertension	0.88 (0.63, 1.21)	0.462
Cystoid macular edema	0.60 (0.31, 1.00)	0.054
Epiretinal membrane	0.92 (0.79, 1.08)	0.500
Cataract	1.00 (1.00, 1.00)	1.000

## Data Availability

The data presented in this study are available on request from the corresponding author.

## Abbreviations

The following abbreviations are used in this manuscript:

RCI	Repository corticotropin injection
IOP	Intraocular pressure
OCP	Ocular cicatricial pemphigoid
ACTH	Adrenocorticotropic hormone
IMA	Immunomodulatory agent
IRs	Incidence rates
CME	Cystoid macular edema
ERM	Epiretinal membrane
IQR	Interquartile range
CI	Confidence interval
RR	Relative risk
